# The Ability of Some Polysaccharides to Disaggregate Lysozyme Amyloid Fibrils and Renature the Protein

**DOI:** 10.3390/pharmaceutics15020624

**Published:** 2023-02-13

**Authors:** Olga Makshakova, Liliya Bogdanova, Dzhigangir Faizullin, Diliara Khaibrakhmanova, Sufia Ziganshina, Elena Ermakova, Yuriy Zuev, Igor Sedov

**Affiliations:** 1 Kazan Institute of Biochemistry and Biophysics, FRC Kazan Scientific Center of RAS, 420111 Kazan, Russia; 2 Chemical Institute, Kazan Federal University, 420111 Kazan, Russia; 3 Zavoisky Physical-Technical Institute, FRC Kazan Scientific Center of RAS, 420029 Kazan, Russia

**Keywords:** lysozyme, amyloid fibrils, disaggregation, polysaccharides, carrageenan, alginate, galactan, chitosan, infrared spectroscopy, atomic force microscopy

## Abstract

The deposition of proteins in the form of insoluble amyloid fibril aggregates is linked to a range of diseases. The supramolecular architecture of such deposits is governed by the propagation of β-strands in the direction of protofilament growth. In the present study, we analyze the structural changes of hen egg-white lysozyme fibrils upon their interactions with a range of polysaccharides, using AFM and FTIR spectroscopy. Linear anionic polysaccharides, such as κ-carrageenan and sodium alginate, are shown to be capable to disaggregate protofilaments with eventual protein renaturation. The results help to understand the mechanism of amyloid disaggregation and create a platform for both the development of new therapeutic agents for amyloidose treatment, and the design of novel functional protein–polysaccharide complex-based nanomaterials.

## 1. Introduction

Many protein aggregation diseases consist of intra- and extracellular accumulation of oligomeric or aggregated proteins [1]. Among different effective inhibitors of unwelcome protein aggregation [2,3,4,5], we want to draw attention to polysaccharides, which can hamper protein amyloidosis, and even repair aberrant proteins [6]. In this work, we extend and exacerbate our research on polysaccharides and protein–polysaccharide systems as the functional basis for novel pharmaceutical applications [7].

Amyloid fibril formation causes a range of malignancies and neurodegenerative diseases [8,9]. Novel treatment ways of such diseases are urgently required and may be developed through a rationale design of peptides and proteins, low molecular drugs [10], or polymeric species [11] slowing down the aggregation and inducing the dissociation of fibrils. In vitro studies using model proteins aim at unveiling the key intermolecular interactions leading to the inhibition of fibrillation and destabilization of protein aggregates, under simplified and controllable conditions. Furthermore, the understanding of the factors that control the protein aggregate integrity paves the way to new bio-based nanomaterials [12,13].

In tissues, amyloidogenic proteins deposit as rigid, non-branching fibrils with a ~10 nm diameter [14]. The cross-β structure is a distinctive feature of amyloid fibrils, as interpreted from X-ray studies [15,16]. Fibril formation can be easily monitored in experiments using, e.g., Congo red dye, which produces an apple-green birefringence when examined between cross polarizers in a light microscope [17], or other specific amyloid molecular probes, such asfluorophore Thioflavin T (ThT) [18]. Kinetics of amyloid formation is, in most cases, characterized by a sigmoidal-shaped time dependence of fibril yield. Such curves are commonly interpreted using crystallization-like models with the nucleation and elongation stages. Thus, the existence of the lag phase can be explained by the slow formation of protein oligomers that play a role in the aggregation of nuclei. The subsequent phase of an exponential rise in fibril conversion is associated with the addition of new protein chains to the fibril nuclei, forming elongated filaments [16]. The filaments may further associate in thicker bunches. For example, the ex vivo fibrils of the human D76H lysozyme isolated from a patient were composed of six circular protofilaments around a helical axis [19]; while in vitro, the hen egg-white lysozyme filaments can assemble into giant multistranded twisted and helical ribbons with a lateral assembly of as many as 17 protofilaments [20].

The amyloid formation may occur from both intrinsically disordered proteins, and misfolded globular proteins [14]. In vitro, fibrils from globular proteins can be obtained when the native protein structure is destabilized in harsh conditions, namely high temperature, low pH and the presence of organic denaturants. In in vivo conditions, charged membranes or other charged surfaces may act as a platform for the assembly of misfolded proteins. Glycosaminoglycans (GAGs) are often found in association with amyloid fibrils isolated from living organisms, implying that they participate in amyloid pathogenesis [21,22]. For example, in hepatic amyloid fibrils, the total amount of glycosaminoglycans was estimated to be 15 μg/mg fibril weight [23]. In Lewy bodies, a hallmark of several neurological diseases, α-synuclein is present together with heparin [24]. Chitin-like polysaccharides were found to be a component of the insoluble Aβ fibrils associated with Alzheimer’s disease [25,26].

Some in vitro studies support the hypothesis that GAGs promote the formation of fibrillar structures. The influence of the polysaccharide structure has also been discussed. Atomic force microscopy (AFM) visualization, ThT fluorescence, CD measurements, and cell viability assays showed that endogenous polysaccharide chondroitin sulfate B promotes the formation of fibrillar structures of the amyloid beta peptide [27]. As revealed by spectroscopic analyses, the fibril formation of the human serum amyloid A is facilitated by GAGs. The role of fibrillation is strongly correlated with the degree of sulfation, but not the backbone structure of GAGs [28]. Heparin and dextran sulfate affected α-synuclein amyloid formation stronger than chondroitin-4-sulfate and dermatan sulfate, which have fewer sulfate groups [29]. Another report showed that four types of GAG molecules (dp 5–18 kDa), namely heparin, chondroitin sulfate A and B and dextran sulfate, revealed different effects on the kinetics of α-synuclein aggregation and the resulting structure and morphology of amyloid fibrils, indicating that both charge distribution and the carbohydrate backbone structure affect the fibril formation [30]. All four GAGs increased the rate of aggregation in a concentration-dependent fashion, except for heparin, which did it only at low concentrations. A sulfated polymer, polyvinyl sulfate, also accelerated α-synuclein aggregation into an amorphous-like globular structure with a lack of typical amyloid fibrillar morphology, however keeping the ThT-binding capability [30].

At the same time, there are numerous examples of fibrillation inhibition by polysaccharides. Positively charged polysaccharide chitosan (at pH 6.5, MW 11.9 kDa) accelerated α-synuclein aggregation kinetics [30], but had an inhibitory effect on Aβ40, which is negatively charged [31]. Chitosan oligosaccharides and chitosan-based nanoparticles inhibited Aβ aggregation and disrupted preformed fibrils in a dose-dependent manner at pH 7.4 [32,33]. Chitosan oligosaccharides also had an inhibitory and disassembling effect on the human islet amyloid polypeptide fibrils [34]. Arabinogalactan protein, a component of gum arabic, was shown to inhibit amyloid fibril formation of bovine insulin and hen egg-white lysozyme, by binding and stabilizing the native state of the proteins [35].

Furthermore, some sulfated polysaccharides, GAG analogues, have an inhibitory effect on amyloid fibril formation [36]. Ascophyllan, a fucose-containing sulfated polysaccharide from brown alga Ascophyllum nodosum, inhibited human insulin fibrillation [37]. Acid fuchsin and its sulfonated triphenyl methyl derivatives were reported to be potent inhibitors of proIAPP(1-48) amyloid formation [38]. Low-molecular-weight heparins can also inhibit fibril formation [21]. Such polysaccharides may play a therapeutic role in vivo, competing for Aβ peptides with proteoglycans that accelerate fibril formation. In general, the effect of polysaccharides on amyloid fibril formation, either promoting the assembly or inhibiting/disassembling the fibrils, depends on the nature of protein and polysaccharide in a rather complex manner.

The success of a transthyretin fibrillation inhibitor, tafamidis, which was clinically approved as a drug against transthyretin amyloidosis, and the discovery of more potent compounds [39] show that the inhibition of fibril formation can be a fruitful approach for the treatment of other fibril-related diseases. An important concern about beta-amyloid aggregation inhibitors is the neurotoxicity of oligomeric Aβ species. Inhibitors binding to the growing aggregate ends slow down the growth stage, but do not affect fibril nucleation, which leads to an increase in oligomer concentration [40]. However, inhibitors binding to the protein monomer inhibit both the nucleation and elongation stages, and are able to disrupt preformed fibrils, as well as toxic oligomers [41]. Such inhibitors should be much safer and are prospective for the development of novel pharmaceuticals. The above-mentioned tafamidis tightly binds to the native transthyretin. Polysaccharides are also often able to bind the monomeric proteins and are very unlikely to be selective for fibril ends. Another important group of inhibitors binding the fibril surface, such as molecular chaperones, slows down the secondary nucleation processes, which results in a significant drop in oligomer concentration [42], despite them being unable to disrupt preformed aggregates.

**Figure 1:**
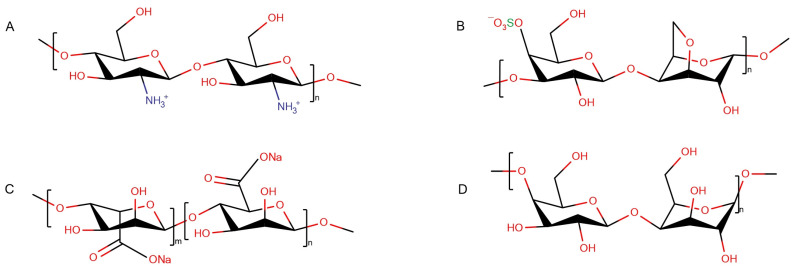
Structural scheme of polysaccharides: chitosan (**A**), κ-carrageenan (**B**), sodium alginate (**C**), β-(1,4)-galactan (**D**).

Here, we compare the effect of various polysaccharides on protein fibrils that were created uniformly (under the same experimental conditions). Hen egg-white lysozyme (HEWL) was used as a model protein [19]. Starting from the thorough characterization of the structural state of fibrils, we show a disaggregating action of some polysaccharides on protofilaments and draw out a generalized conclusion about key factors regulating this process. The structural organization of fibrils was characterized using Fourier transform infrared (FTIR) spectroscopy and AFM. The polysaccharide range comprised: linear positively charged chitosan (Figure 1A), linear negatively charged κ-carrageenan (Figure 1B) and sodium alginate (Figure 1C), as well as neutral β-(1,4)-galactan (Figure 1D).

The results shed light on the of protein–polysaccharide interactions and create a platform for the development of macromolecules/approaches regulating protein folding, which can be used as chaperons in pharmacology and as low-cost, environmentally friendly bio-based materials in the industry of polymers [43,44].

## 2. Materials and Methods

### 2.1. Materials and Fibril Preparation

Lyophilized HEWL and polysaccharides were purchased from Sigma-Aldrich and used without preliminary purification: hen egg-white lysozyme (lot L6876), chitosan (lot 448877), κ-carrageenan (lot 22048) and sodium alginate (lot 180947). The β-(1,4)-galactan, a polysaccharide of the rhamnogalacturonan I side chains, was purchased from Megazyme (P-GALPOT, 120501c).

HEWL fibrils were prepared by incubating 5 mL of 15 mg/mL^−1^ (1 mM) protein solution containing 25 mM NaCl and 10 mM HCl at pH 1.9 at 65 °C in a thermostat, with stirring. The solution was kept under the described conditions for 5 days. The growth of the fibrils was confirmed using spectrofluorimetry, with a thioflavin T (ThT) fluorescent indicator.

Mature fibrils were dialyzed against pure 400 mL of distilled water, and the solution was changed after 2, 4, 6, 8, and 24 h (total volume used amounted to 2 L). The quality of the dialysis was monitored by the electrical conductivity of the dialysate. The convergence of conductivity changes was considered as the complete removal of salt excess. Then dialyzed samples were centrifuged. The water-soluble fraction was used for further studies. The concentration of the water-soluble fraction of protein fibrils was controlled by UV adsorption intensity at 280 nm (extinction coefficient ε_280_ = 2.65 mg/mL^−1^/cm^−1^) [45].

### 2.2. Monitoring Fibril Growth Using a ThT Fluorescence Assay

A 50 μM solution of ThT was prepared in a 25 mM phosphate buffer at pH 7.4. The samples of incubated protein were added to cuvettes containing ThT solution with buffer. The final concentrations of protein and ThT were 3 and 10.4 μM, respectively. The cuvettes were kept for 10 min at 25 °C, then fluorescence spectra were recorded using a Cary Eclipse fluorescence spectrophotometer (Agilent Technologies, Santa Clara, CA, USA) at the emission wavelength range of 460 to 500 nm, λex = 450 nm.

### 2.3. Preparation of HEWL Fibril–Polysaccharide Mixtures

Aqueous solutions of κ-carrageenan and sodium alginate were allowed to swell for one hour at 20 °C and were then stirred for two hours at 70 °C. Galactan and chitosan were dissolved at room temperature. To dissolve chitosan, hydrochloric acid was added to pH = 4.

The polysaccharide solutions were mixed with the supernatant fraction of HEWL fibrils in a mass ratio polysaccharide to protein of 1:3, as in ref. [6], which is sufficient for a charge compensation with negatively charged polysaccharides within the conditions used.

We worked with the light fractions appearing in supernatants and monitored the changes in structural organization and the size of the fibrils. Insoluble fibrillar sediments are not appropriate for the analysis because of several reasons: strong light scattering hampering the use of optical methods, difficulty to control the protein/polysaccharide ratio, and difficulty to prepare the homogeneous mixture. Another interfering factor is a high salt concentration in the buffer solution, which also leads to light scattering, aberrant absorption and salt crystallization upon drying on a surface, hiding the molecular shape of fibrils in AFM. Therefore, the excess salt was removed by dialysis and the light fraction of fibrils was used for further analysis of fibril–polysaccharide interactions. The final pH of the mixtures was controlled by a pH meter and the samples were titrated to pH 7.0, when needed.

### 2.4. CD Spectroscopy

The supernatant fraction of the HEWL fibrils was diluted with the phosphate buffer (NaH_2_PO_4_, Na_2_HPO_4_, 0.25M, pH = 7.0) to a final protein concentration of 0.05 mg/mL^−1^. The spectra were registered in the wavelength range of 190–280 nm, using a Jasco J-1500 circular dichroism spectrophotometer under stirring at 30 °C. The spectra were analyzed using the CDSSTR method available via the DichroWeb online service [46].

### 2.5. AFM Measurements

The fibril samples were diluted to the final protein concentration of 0.15 mg/mL^−1^ (10 μM with respect to the monomeric protein). A 10 μL droplet was deposited onto freshly cleaved mica. After 1 min incubation, the mica disk was rinsed with Milli-Q water and dried with a weak stream of nitrogen. AFM experiments were performed on the dried sample using a Titanium microscope (NT-MDT, Russia). Measurements were carried out in a tapping mode in air atmosphere at room temperature. Cantilevers NSG-11, with a force constant of 2.5–10.5 N/m and resonance frequency of 115–190 kHz (NT-MDT, Russia), were used.

### 2.6. FTIR Spectroscopy

The FTIR spectra were recorded using the IRAffinity-I spectrometer (Shimadzu, Europa GmbH, Duisburg, Germany), equipped with the attenuated total reflection (ATR) accessory, with a ZnSe crystal. The spectra were recorded at 4 cm^−1^ resolution in the 4000–800 cm^−1^ wavenumber range. For each spectrum, 256 scans were accumulated.

The samples of the solution and gels were placed on the surface of an ATR-sensing element and thermostat at 25 °C. The spectra of the solutions were recorded in their hydrated state and as dry films. Films were prepared by in situ solvent evaporation at the same experimental conditions. Drying was aimed at getting rid of the interfering influence of water absorbance in the amide I region sensitive to the protein secondary structure. All spectra were subjected to both water vapor and liquid water absorbance compensation.

FTIR spectroscopy is a unique method to probe the secondary structure of proteins and protein–polysaccharide mixtures in different states, including gels, since these two types of biopolymers absorb in different spectral regions on the FTIR spectrum [47,48,49].

## 3. Results

### 3.1. Characterization of Water-Soluble HEWL Fibrils

**Figure 2:**
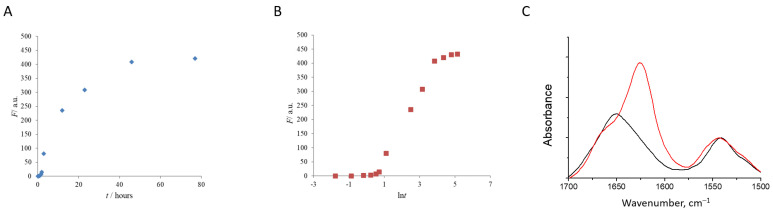
The kinetic curve of the HEWL fibril growth (at pH = 1.9 and T = 65 °C) followed by ThT fluorescence intensity (**A**,**B**). Relative FTIR absorbance spectra show the conversion of native HEWL (black) into fibrils (red) (**C**).

Figure 2A,B depict a lysozyme fibril growth profile as followed by ThT fluorescence intensity. The kinetic curve of the HEWL fibrillation has a sigmoidal shape. It comprises a lag phase (about 2 h) linked with the formation of nuclei, a fibril elongation phase corresponding to the active fibril growth (completing in about 50 h), and a plateau, at which further incubation of the protein does not lead to the growth of fluorescence intensity. Similar curves have been observed in other studies of HEWL fibrils [50,51]. In literature, many different protocols of fibril growing can be found [52,53,54]. The exact kinetic parameters of fibril formation are heavily dependent on experimental conditions, such as temperature, protein concentration, stirring rate, or the presence of other substances [55,56].

The FTIR spectra can be used to distinguish between the parallel cross-β-structure of the amyloid fibrils and the antiparallel beta-structure of the protein aggregates [57,58,59]. The former is characterized by the peak at 1620–1630 cm^−1^, and the feature of the latter is an additional sharp satellite band at 1695 cm^−1^ [57,60]. The FTIR spectrum of the HEWL fibrils revealed the dominant parallel beta structure with the main FTIR peak at 1625 cm^−1^ and a minor band at 1668 cm^−1^ (Figure 2C). FTIR spectroscopy is sensitive to short-range interactions and reflects the formation of the parallel β-structure-rich unfolded state [61].

**Figure 3:**
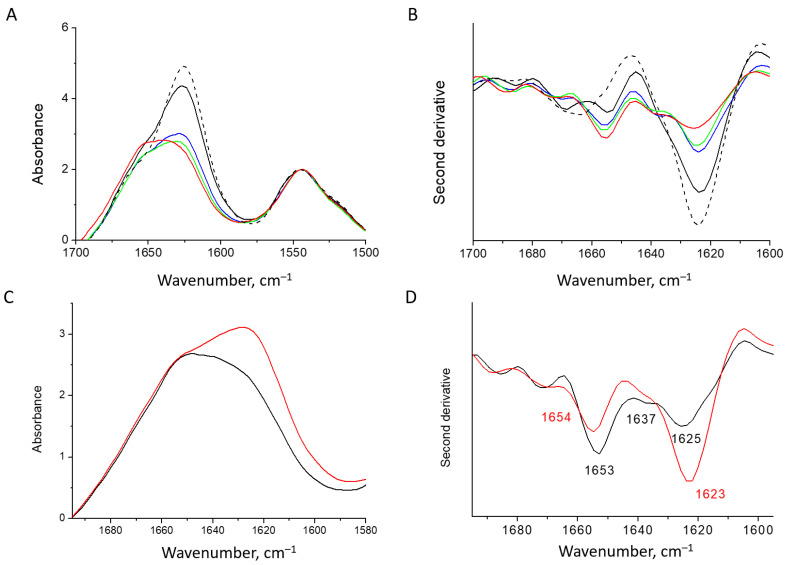
Absorbance spectra (**A**) and second derivative spectra (**B**) of the HEWL fibrils at 25 °C: dashed line—initial mature fibrils; black, blue, green and red—fibrils dialyzed against water for 2, 4, 6 and 24 h, respectively. Absorbance spectra (**C**) and second derivative spectra (**D**) of two fractions of the HEWL fibrils separated by centrifugation: sediment—red and supernatant—black.

The presence and concentration of salts are important for the HEWL fibril formation, and determine whether the protofilaments develop into mature amyloid fibrils or other types of aggregates [62]. In the presence of inhibitors of fibrillation, the ions counteract inhibiting effects toward amyloidogenesis [63]. We found that removing salt from the HEWL fibril solution leads to the redistribution of FTIR intensity. The intensity of the band at 1625 cm^−1^ gradually decreases over time and the intensity of the 1655 cm^−1^ increases (Figure 3A,B). The appearance of minor bands at around 1655 cm^−1^ and 1668 cm^−1^, along with 1625 cm^−1^ for the mature fibrils, is similar to the observations reported for human lysozyme fibrils [64]. Interestingly, separation of the sample upon centrifugation shows that the heavier fraction has a stronger β-structure, whilst in the spectrum of the light fraction of supernatant, the band at 1655 cm^−1^ is dominant and the band at 1625 cm^−1^ is less pronounced (Figure 3C,D).

In HEWL fibrils, the band at 1655 cm^−1^, rising with the desalination, resembles the main band of the native lysozyme, attributed to its helical structure [65]. Upon drying, this band significantly broadens and shifts contrary to the behavior of the native protein spectrum (Figure 3C,D). Therefore, we can attribute this band to the destabilized helical elements. The CD spectra of HEWL fibrils in the supernatant support the conclusion that along with β-structure, there is a large portion of the α-helical structure. The spectral shape deconvolution reveals the presence of 35% of α-helices, 28% of β-structures, and 37% of unordered structures, including β-turns (Figure S1). This is similar to the secondary structure composition in human lysozyme amyloid fibrils [65].

**Figure 4:**
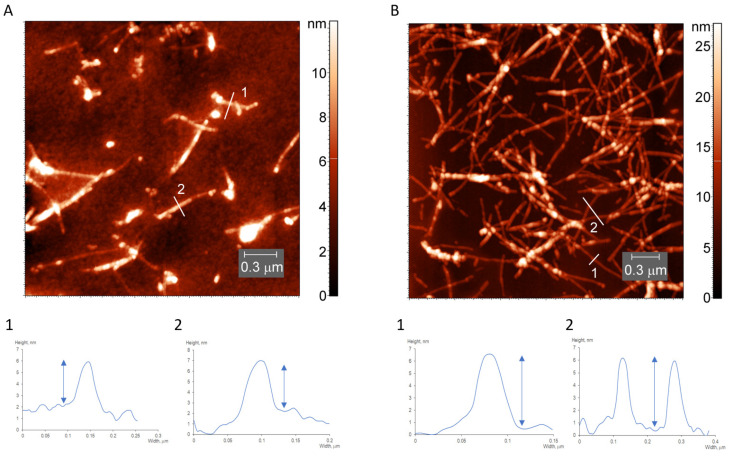
AFM images and height profiles of the light (**A**) and heavy (**B**) fractions of desalted lysozyme fibrils.

In the light water-soluble fraction, the AFM image reveals the presence of long unbranched fibrils with a height of ~4 nm, width of ~10 nm and several nanometers in length (Figure 4A). In the heavy-water fraction, which appeared as a precipitate, the fibrils are thicker, with a minimal height of ~6 nm (Figure 4B). This is in line with the dimensions reported in the literature. The single filaments were reported to have a constant height of 3.7 ± 0.4 nm and a width of 10 ± 0.3 nm [20]. In another report the size of protofibril was estimated, using AFM, to be 3.9 ± 0.2 nm in height and 9.6 ± 1.0 nm in width [66]. The dimensions of mature fibrils were reported to be 6.0 ± 0.2 nm in height and 13.9 ± 1.0 nm in width [66]. Therefore, we conclude that we have mature fibrils in precipitate and protofilaments in the soluble fraction.

Summing up, our data revealed that the light fraction of HEWL fibrils in the supernatant is represented by long unbranched protofibrils. Washing out the salt excess increases the number of disordered elements in the fibrils and does not affect the quality of the parallel β-structure.

### 3.2. HEWL Fibril–Polysaccharide Mixtures

Polysaccharide charge and its accessibility for the protein have a decisive role in phase behavior and conformational transformations of the HEWL fibrils when they are mixed with polysaccharides. Interactions of two linear negatively charged polysaccharides, κ-carrageenan and sodium alginate, with HEWL fibrils, lead to phase separation with gel-like matter sedimentation. In contrast, weaker interactions with β-(1,4)-galactan allow the protein–polysaccharide complexes to remain soluble. Lysozyme fibrils mixed with a positively charged linear polysaccharide, chitosan, also remain soluble.

#### 3.2.1. HEWL Fibril–Chitosan Mixtures

**Figure 5:**
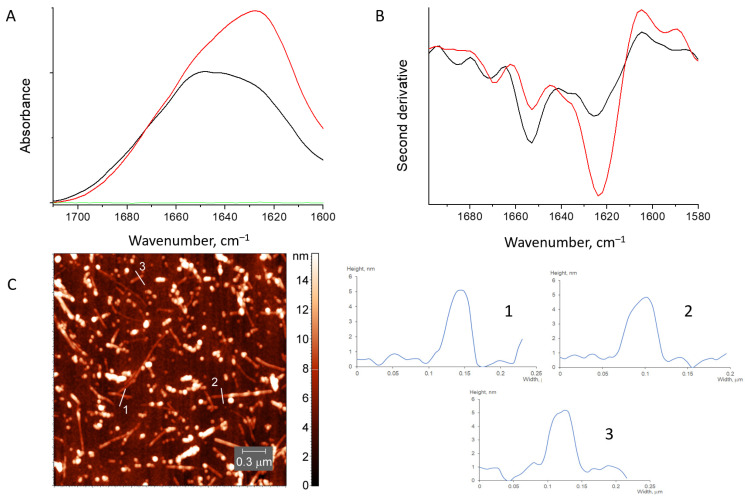
Absorbance spectra (**A**) and second derivative spectra (**B**) of HEWL fibrils mixed with chitosan (red), initial HEWL fibrils (black) and pure chitosan (green). AFM image of HEWL fibrils mixed with chitosan (**C**).

AFM images show that upon mixing with chitosan, some long HEWL fibrils, with the height of about 4 nm, remain. Beside them, oligomers of a smaller length appeared (Figure 5). FTIR spectra of the solution illustrate the retention and even intensification of the band at 1623 cm^−1^ resulting from the parallel β-structure. An increase of the band intensity occurs at the expense of the band at 1655 cm^−1^ resulting from α-helical elements of structure (Figure 5). The spectra decomposition, together with the estimation of the secondary structure content, is given in Supplementary Materials (Figure S2 and Table S1).

Therefore, both methods support the presence of fibrils in the mixture.

#### 3.2.2. HEWL Fibril–β-(1,4)-Galactan Mixtures

**Figure 6:**
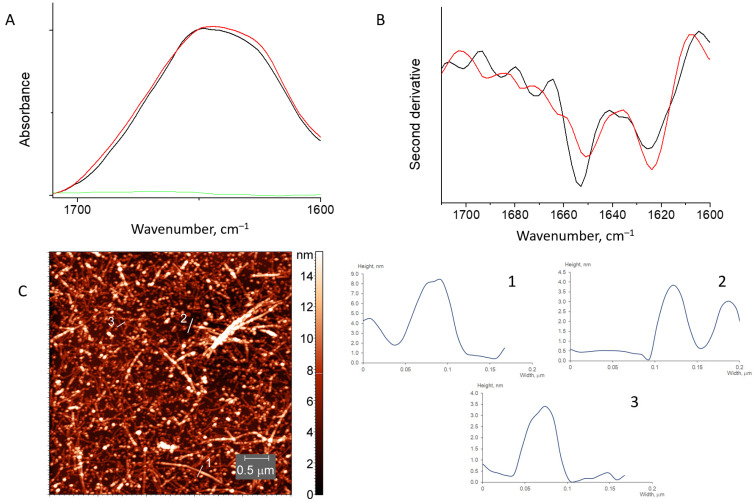
Absorbance spectra (**A**) and second derivative spectra (**B**) of HEWL fibrils mixed with β-(1,4)-galactan (red) and pure HEWL fibrils (black). The spectrum of β-(1,4)-galactan is in green. AFM image of HEWL fibrils mixed with β-(1,4)-galactan (**C**).

AFM images of the mixtures of HEWL with galactan also revealed the presence of long fibrils, together with the appearance of oligomers of a round shape. In contrast to chitosan, partial fibril defragmentation by galactan is accompanied by less pronounced alternation of the HEWL FTIR spectrum (Figure 6). The FTIR spectrum of galactan + HEWL fibrils mixtures is similar to that of pure HEWL fibrils. The amide I region of the spectrum revealed the amyloid β-sheets (bands at 1623 cm^−1^) in similar amounts as in the spectrum of pure fibrils (Figure 6 and Table S1). The content of α-helices (band at 1655 cm^−1^) decreases with the proportional increase of the content of random coils (band at 1663 cm^−1^, see Figure S2), implying their interconversion.

#### 3.2.3. HEWL Fibril–κ-Carrageenan Gels

**Figure 7:**
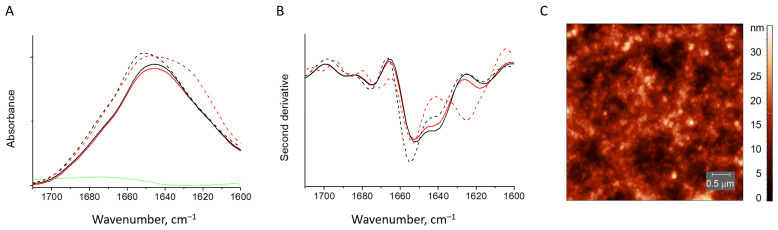
Absorbance spectra (**A**) and second derivative spectra (**B**) of HEWL fibrils (dashed red line) and those mixed with κ-carrageenan (solid red line), native HEWL mixed with κ-carrageenan (solid black line), native HEWL (dashed black line). AFM image of the trace from the gel formed by HEWL fibril and κ-carrageenan mixture (**C**).

When HEWL fibrils are mixed with κ-carrageenan, the gel coacervate is formed. A similar behavior was reported for the native lysozyme mixed with κ-carrageenan. In such gels, the ratio of protein and polysaccharide equals 3:1 and is governed by the charge compensation. Lysozyme adjusted its structure by an increase of the native β-structure content [6]. When fibrillar HEWL interacts with κ-carrageenan, the protein fully renatures. The band at 1623 cm^−1^ fully disappears and the spectrum becomes identical to that of the native HEWL–κ-carrageenan gels (Figure 7A,B and Figure S2).

The AFM images obtained for the traces of gel sticking to the mica after its removal show no fibrils in the volume of the gel (Figure 7C). The same was true for the supernatant (the liquid not entrapped into the gel network).

#### 3.2.4. HEWL Fibril–Sodium Alginate Gels

**Figure 8:**
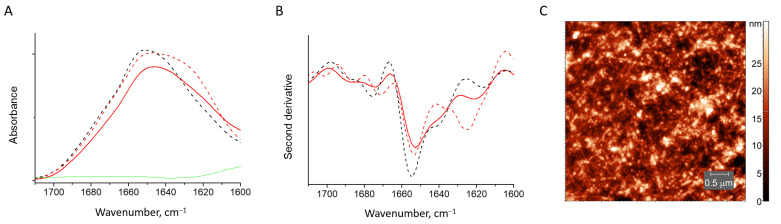
Absorbance spectra (**A**) and second derivative spectra (**B**) of HEWL fibrils (dashed red line), those mixed with sodium alginate (solid red line), native HEWL (black dashed line) and pure sodium alginate (green). AFM image of HEWL fibrils mixed with sodium alginate (**C**).

The mixtures of HEWL fibrils with sodium alginate revealed a behavior similar to the mixtures with κ-carrageenan. The gel is precipitated. In the FTIR spectrum, there is no sign of fibrils. The spectrum of the protein is close to the spectrum of the native HEWL bound to the sodium alginate, with an increase of the absorbance at 1640 cm^−1^ when compared to the native HEWL spectrum (Figure 8A,B). Therefore, apparently, there are no particular effects from the type of polysaccharide backbone, the type of negatively charged groups and the distance between them, on the disaggregation and renaturation of HEWL fibrils. Both κ-carrageenan and sodium alginate bind HEWL fibrils and convert them into native-like protein. The AFM image is also similar to that of the protein-κ-carrageenan gel, revealing no fibrils in the volume of the alginate gel (Figure 8C).

It is important to note, that the disaggregating effect of the linear negatively charged polysaccharides was detected only for the light fraction of desalted fibrils. The sedimented fibrils also formed gels with both κ-carrageenan and alginate, but they remained fibrillar and rich in parallel β-structure. The presence of helical elements in the light fraction of the desalted HEWL fibrils could be responsible for easier disaggregation and renaturation.

## 4. Discussion

**Figure 9:**
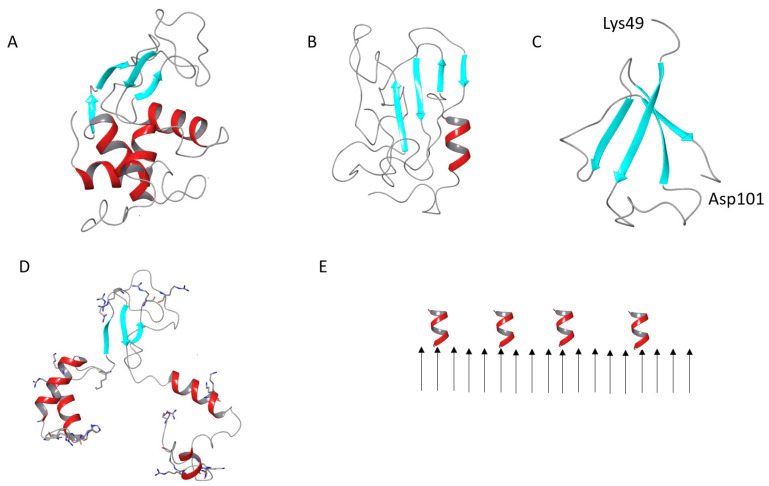
Cartoon representation of the native HEWL (**A**), a model of the β-structure-rich conformer of denatured HEWL (**B**), a model of the β-structure-rich conformer of amyloidogenic HEWL fragment (**C**), a model of the partially desaturated HEWL globule, positively charged residues are shown in sticks (**D**), a scheme of the deposition of helices on the HEWL fibril surface (**E**).

A lysozyme is a classical protein model for amyloid research [19]. Deposition of the human lysozyme as amyloid fibrils occurs in some forms of systemic nonneuropathic amyloidosis. Human lysozymes depose as amyloid fibrils in the kidney. Some point mutations accelerate this deposition [14]. The HEWL is structurally close to the human lysozyme, having the same fold (Figure 9A) and 87% primary structure similarity. The HEWL can be used to study the inhibitory action of antibodies or small molecules on its aggregation, aiming at transferring the findings to therapeutic applications for the treatment of amyloidosis [19]. In the present work, we compared how various polysaccharides differ in the effect on lysozyme fibrils, and figured out the prerequisites for the fibril disaggregation and HEWL renaturation. The analysis revealed two key findings: 1. polysaccharide charge is important, as negatively charged polysaccharides were capable of renaturing HEWL from fibrils; 2. the fine structure of HEWL fibrils is important, as protofibrils containing α-helices were responsive to the polysaccharide action.

To suggest any hypotheses about the mechanism of polysaccharide action, the structure of HEWL molecules in fibrils should be clarified. According to numerous reports about the fold of lysozyme molecules in amyloid fibrils, the parallel β-sheets are complemented by α-helical and random coil elements. In human lysozyme fibrils, the fraction of β-sheets was estimated to be 79%, while α-helical/random structure amounted to 14% [64]. Booth et al. [65] also detected the presence of helical structure in the human lysozyme amyloid fibrils, using FTIR spectroscopy. However, not seeing any sign of helices in X-ray diffraction patterns of fibrils, they assumed that the helical elements might not be regularly ordered. In addition, the authors suggested the following fibril structure: β-domain (about 35% of the native protein sequence) forms an amyloid core, while the α-domain, comprising fragments at N- and C-termini, remains out of the fibril core and may retain its helical conformation [65]. The results of the present study highlight the dependence of the stability of helices on ionic strength. Removing the salt excess returns conformational flexibility and the possibility to form helices (up to 35%). This is an argument in favor of the surface location of the helix-forming residues, in agreement with the hypothetical structure described above.

Some attempts to shed light on the atomistic structure of the HEWL molecules in fibrils were made using computational studies [67,68]. In addition, recently, we reported the results of an accelerated molecular dynamics (aMD) study on the capability of the HEWL molecule to form β-structure-rich intermediates upon denaturation [69]. The enhanced sampling of the conformational landscape revealed a number of clusters with stable structures, dominated by β-sheets. Some of these structures also evidenced the tendency of the terminal fragments to form α-helices (Figure 9B). However, the β-structure formed by the chains of a single-protein molecule is predominantly anti-parallel, which is not typical for protein units in amyloid fibrils.

Not only can a whole HEWL molecule form fibrils, but its amyloidogenic fragment, encompassing residues 49 to 101, can also do this [70]. The amyloidogenic fragment partially intersects with the β-domain, which includes residues 39 to 85 [71]. The aMD simulations revealed a number of β-structure-rich conformations of a single amyloidogenic peptide molecule, while several molecules can favorably aggregate [72]. Some antiparallel β-structure was also observed (Figure 9C). The fibril nuclei with parallel β-structure are formed from various conformations capable of aggregating during the pre-nucleation phase [59].

Nonetheless, in the absence of the atomic resolution structure of fibrils, the models described are useful to compare the size of aggregates with AFM data. In native HEWL, the β-domain has the dimensions of around 3 × 2 × 2 nm. If it is fully extended for the side-by-side assembly, as proposed by [65], the length would be around 10 nm and the helix-forming fragments would be protruding from the propagating β-sheet by ~5 nm from each side. This is larger than the AFM height of protofibrils (~4 nm). AFM provides accurate height data, while width dimensions may be larger due to a finite tip–width convolution error [73]. The model of the full-length HEWL converted into a β-rich structure (Figure 9B) has the dimensions of 4 × 3 × 2.5 nm. The model of an amyloidogenic fragment (Figure 9C) gives 3.5 × 3.2 nm. The additional helices, of which the length is redistributed, would be still quite long, at ~4 nm on the C-terminus and ~6 nm on the N-terminus, and would reside on the opposite sides of the core of the fibril. However, since helices do not have a regular structure, they would not contribute to the regular dimensions seen by AFM. Being flexible, the helices may perturb upon drying, as evidenced by our FTIR experiments; therefore, they might be only partly visible by AFM.

Intriguingly, positively charged residues are decisive in lysozyme amyloid formation. Acetylation of the lysine residues promotes amyloid formation. In contrast, when citraconyl groups are linked to the free lysine groups, in order to switch their charge to negative (at a neutral pH), HEWL aggregation at acidic pH is inhibited [74]. The importance of salt for fibril formation and its development into mature amyloid fibrils [62] points to the necessity to screen the charge for proper assembly. The fact that amyloid fibrils restore a major part of α-helices (35% in our experiment), upon washing out the salt, evidences the surface location of charged residues tending to repulse and detach from the core of the fibril. This is in the agreement with the above-discussed hypotheses about nanoscale fibril organization. The densely packed, positively charged residues are predominantly located in α-domain: seven at the N-terminus and seven at the C-terminus (Figure 9D). In these regions, the two groups of the basic residues K1, R5, R21, K13, R14, H15, K33 and K96, K97, R112, R114, K116, R125, R128 have the potential to interact with the negatively charged polysaccharides and trigger the fibril disassembly. Only four positively charged residues are located in the β-domain. Recently, we demonstrated that the charge density on κ-carrageenan (differing for its conformations) determines the binding epitope on the native HEWL surface. Namely, the double helix of κ-carrageenan preferentially binds to the positively charged spots in the α-domain and a single chain of the κ-carrageenan binds to the β-domain [6]. The results of the current study highlight that the location (hence, availability) of positively charged residues on the surface of HEWL fibrils is crucial for establishing appropriate contacts with negatively charged polysaccharides and the consequent fibril dissociation and renaturation. The surface location of the helices on the HEWL fibrils is schematically shown in Figure 9E.

Provided that positively charged, flexible fragments of the HEWL fibrils are available for interactions with polysaccharides, we observed three types of polysaccharide effects on the HEWL fibril structure. The effect of the first type was from the neutral β-(1,4)-galactan. This polysaccharide was not capable of dissolving fibrils. Despite the fibril defragmentation being visible, the thin structure of the protein changed slightly, namely a part of the helical conformation (~10%) was converted into the coil structure. This may be due to weak interactions of the helices located on the fibril surface with neutral polysaccharide. The content of the amyloid β-structure remained almost the same as in initial fibrils. The effect of the second type was from the positively charged chitosan. This polysaccharide, similar to galactan, was not capable of disrupting and dissolving HEWL fibrils, revealing only partial defragmentation of the fibrils. However, in contrast to galactan, the structure of the HEWL in fibrils changed significantly. The main feature of the polysaccharide effect was the increase in the content of amyloid β-structure and the decrease in the number of α-helices. Presumably, the positively charged groups of chitosan resulted in the protein charge screening/repulsion with the re-establishment of homogeneous β-structure of fibrils. The third type of polysaccharide effect was from negatively charged polysaccharides, κ-carrageenan and sodium alginate. Interactions with these polysaccharides resulted in the full disappearance of the hallmarks of the amyloid β-structure presence and in the renaturation of native protein structure. We assume the latter finding is an important fundamental base for pharmaceutical applications. The defragmentation effect of neutral and positively charged polysaccharides on fibrils seems to be less prospective, since oligomers can be toxic.

Our findings can be also compared with the disassembling effect of functionalized metallic (gold and silver) nanoparticles on the lysozyme amyloid fibrils studied previously [75,76,77], and also attributed to their electrostatic interactions with the protein. Negatively charged silver and iron nanoparticles were reported to sequester monomers of the positively charged human islet amyloid polypeptide and inhibit fibril formation [78]. The solubilizing and disaggregating effect was reported for sulfated and sulfonated polymers [79]. It was also stated that for better disrupting amyloid aggregates, the polyelectrolytes should be tightly bound [80]. The results of our research support the latter finding, since in gels, long polysaccharides, κ-carrageenan and sodium alginate, tightly interact with the protein (according to mass ratio, 12 disaccharides were bound to one protein molecule). It is yet to be studied to what extent negatively charged polysaccharides are capable of disaggregating fibrils and renaturing protein.

Another finding of the current research is that the HEWL mature fibrils mostly have β-structure and do not have α-helices that cannot be destroyed by polysaccharides. On the one hand, this shows the limitation of the destructive effect of polysaccharides on fibrils and their potential therapeutic applicability. On the other hand, this fundamental knowledge is important for the rational design of cross-β based biomaterials [81], amyloid-based nanoscaffolds [82] and protein–polysaccharide-based materials, where polysaccharides are used to reinforce amyloid fibril gels [43].

## 5. Conclusions

Thus, the structure of in vitro fibrils, including the accessibility of charged groups, can be crucial for fibril stability and their use in technology. The potential of natural polysaccharides and their synthetic fine-tuned analogues to disaggregate amyloid fibrils is highlighted, but the precise mechanism is yet to be unveiled.

In this study we demonstrated that linear polysaccharides have a different effect on fibril desegregation, strongly dependent on their charge. The neutral β-(1,4)-galactan introduces subtle changes in the structure of fibrils. The positively charged chitosan improves the quality of cross β-structure. Both polysaccharides do not fully disaggregate fibrils. However, the interaction of linear, negatively charged κ-carrageenan and alginate with HEWL fibrils results in the protein refolding conversion into the native-like structure.

We conclude that positively charged helices on the surface of HEWL filaments and their interactions with polysaccharides are decisive for protein renaturation. The following model was suggested to explain our data on HEWL fibrils: polysaccharide interactions. In fibrils, the positively charged helical fragments belonging to the α-domain in the native HEWL reside out of the core of fibrils. If placed on the surface of the fibril, they may form a ‘hairy’ region, accessible to polysaccharides. In a single chain of a protofibril, the negatively charged polysaccharides, κ-carrageenan and alginate, interact with positively charged helices on the HEWL fibrils, and repair initial protein folding. The complex formation is driven by charge compensation, since the gel can be dissolved by adding an extra amount of salt. In such gel, the protein undergoes structural modification with the increase of β-structure, typical for the native protein (with an FTIR absorbance at 1640 cm^−1^). In the current study, we also observed a full precipitation of HEWL happening upon the addition of 3:1 (by mass) carrageenan. In the resulting gel, the protein was renatured. The results help to understand the mechanism of amyloid disaggregation and create a platform for both the development of new therapeutic agents for amyloidoses treatment, and the design of novel functional protein–polysaccharide complex-based nanomaterials.

## Data Availability

The data in this study are available on reasonable request from the corresponding author.

## Supplementary Materials

The following supporting information can be downloaded at: https://www.mdpi.com/article/10.3390/pharmaceutics15020624/s1, Figure S1. CD spectra of native HEWL (black) and soluble fraction of HEWL fibrils (red). Figure S2. Second derivative of FTIR spectra: insoluble fraction of HEWL fibrils (a), soluble fraction of HEWL fibrils (b), soluble fraction of HEWL fibrils mixes with chitosan (c), soluble fraction of HEWL fibrils mixes with galactan (d), soluble fraction of HEWL fibrils mixes with κ-carrageenan (e), soluble fraction of HEWL fibrils mixes with sodium alginate (f). Table S1. Some characteristics of main components of FTIR spectra.
