# Supplementary material for: The Ability of Some Polysaccharides to Disaggregate Lysozyme Amyloid Fibrils and Renature the Protein
Source: Pharmaceutics. 2023 Feb 13;15(2):624. doi: 10.3390/pharmaceutics15020624 (PMC9962556; doi:10.3390/pharmaceutics15020624)
Supplement: Supplementary file 1 [file pharmaceutics-15-00624-s001.zip › pharmaceutics-2139896-supplementary.pdf]

pharmaceutics

Supplementary Materials: Novel polysaccharide-protein systems as a fundamental base for anti-amyloid pharmaceutical applications

Olga Makshakova, Liliya Bogdanova, Dzhigangir Faizullin, Diliara Khaibrakhmanova, Sufia Ziganshina, Elena Ermakova, Yuriy Zuev and Igor Sedov

CD spectra of native HEWL and soluble fraction of HEWL fibrils

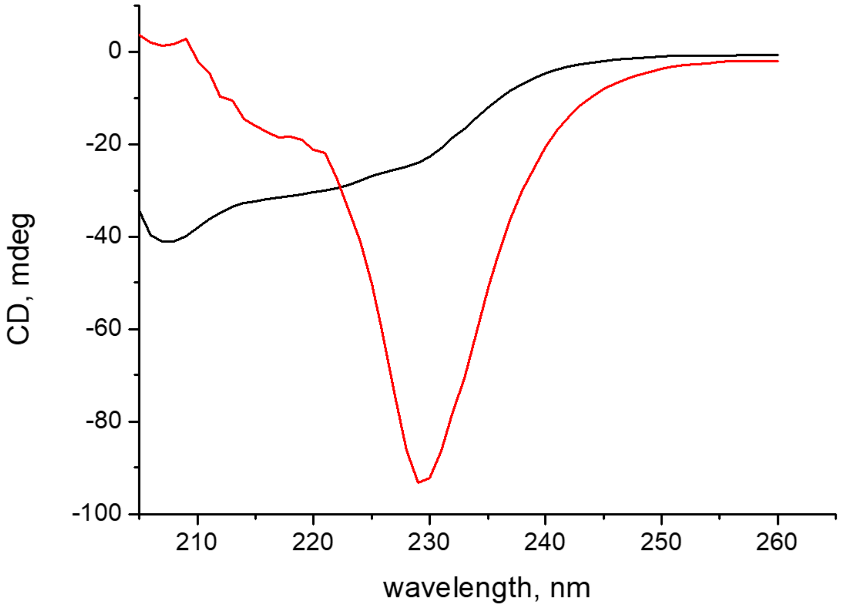

Figure S1. CD spectra of native HEWL (black) and soluble fraction of HEWL fibrils (red).

FTIR spectra of HEWL fibrils initial and upon interactions with polysaccharides

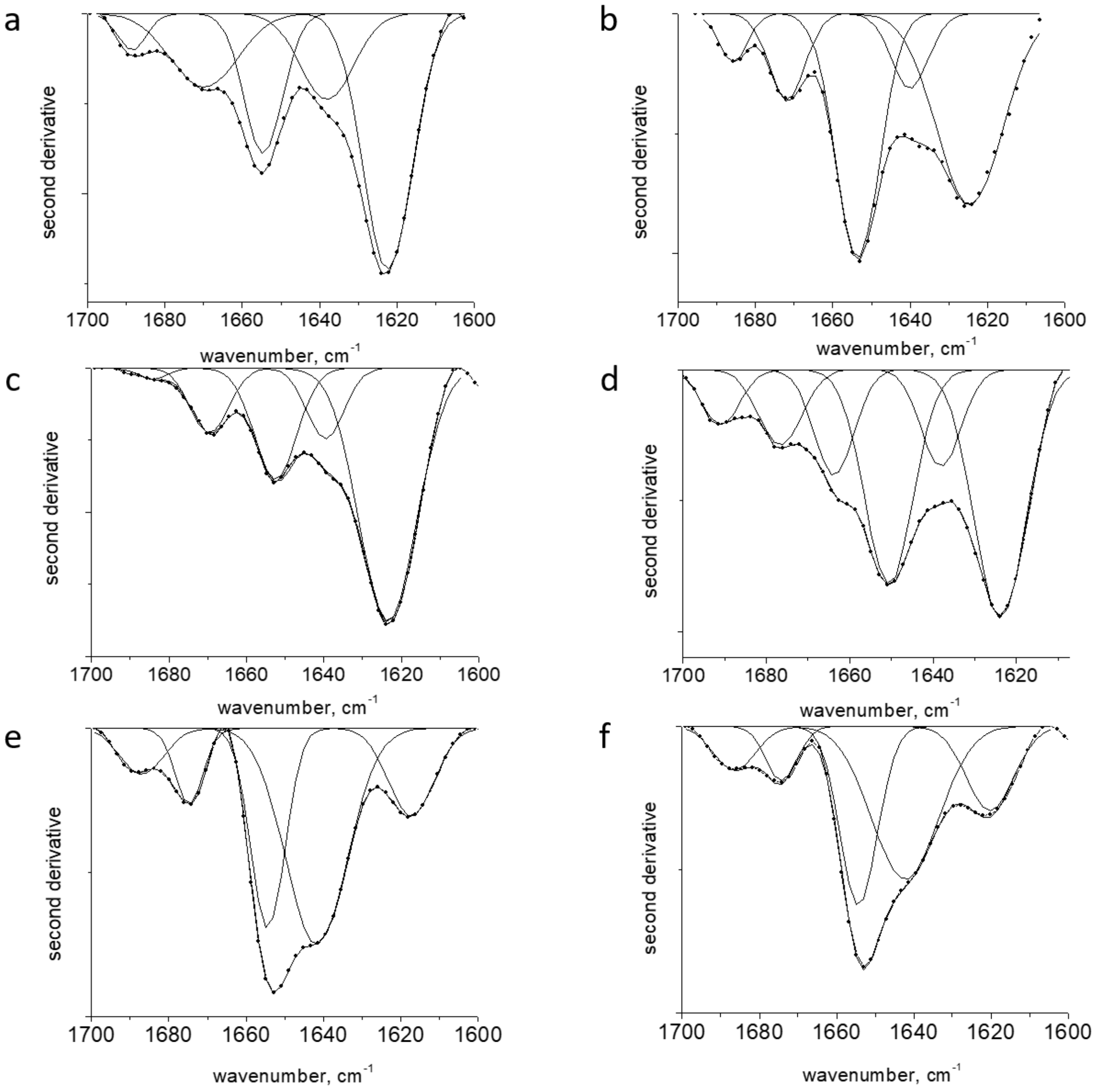

Figure S2. Second derivative of FTIR spectra : insoluble fraction of HEWL fibrils (a), soluble fraction of HEWL fibrils (b), soluble fraction of HEWL fibrils mixes with chitosan (c), soluble fraction of HEWL fibrils mixes with galactan (d), soluble fraction of HEWL fibrils mixes with κ-carrageenan (e), soluble fraction of HEWL fibrils mixes with sodium alginate (f).

Table S1. Some characteristics of main components of FTIR spectra. .

Sample	Wavenumber, cm^{-1}	Halfwidth, cm^{-1}	Peak area, %	Peak attribution
I	1622	15	44	
S	1624	20	40	
Ch	1623	17	58	amyloid β-structure
G	1623	15	36	+ side chains*
C	1617	14	14	
A	1620	14	16	
I	1654	12	19	
S	1653	13	35	
Ch	1652	13	19	
G	1651	14	28	α-helix
C	1654	10	24	
A	1654	11	27	
I	1638	16	16	
S	1640	9	9	
Ch	1640	10	11	intramolecular
G	1638	10	11	β-structure
C	1642	18	47	
A	1642	20	42	

* the contribution of side chains clearly appearing for C and A was not subtracted from the spectra of I, S, Ch and G. The area of whole band was taken to estimate the content of β-structure. Samples denoted as: I – insoluble fraction of HEWL fibrils, S – soluble fraction of HEWL fibrils, Ch – soluble fraction of HEWL fibrils mixes with chitosan, G – soluble fraction of HEWL fibrils mixes with galactan, C – soluble fraction of HEWL fibrils mixes with κ-carrageenan, A – soluble fraction of HEWL fibrils mixes with sodium alginate.
